# Efficacy of hyperbaric oxygen therapy for diabetic foot ulcer, a systematic review and meta-analysis of controlled clinical trials

**DOI:** 10.1038/s41598-021-81886-1

**Published:** 2021-01-26

**Authors:** Rakesh Sharma, Suresh K. Sharma, Shiv Kumar Mudgal, Prasuna Jelly, Kalpana Thakur

**Affiliations:** 1 College of Nursing, All India Institute of Medical Sciences, Rishikesh, Uttarakhand India; 2 Akal College of Nursing, Eternal University, Baru Sahib, Himachal Pradesh India

**Keywords:** Cell biology, Endocrinology, Medical research

## Abstract

Studies have suggested that hyperbaric oxygen therapy (HBOT) is effective in the healing of diabetic foot ulcer (DFU); however, there is a lack of consensus. Therefore, to assess the efficacy of HBOT on diabetic foot ulcer among diabetic patients, controlled clinical trials were searched through PubMed, EMBASE, Clinical key, Ovid Discovery, ERMED, Clinical Trials.gov databases for randomized controlled trials (RCTs) and other sources until 15 September 2020. Studies that evaluated the effect of HBOT on diabetic foot ulcer, complete healing, amputation, adverse events, ulcer reduction area, and mortality rate were included. Of 1984 study records screened, 14 studies (768 participants) including twelve RCTs, and two CCTs were included as per inclusion criteria. The results with pooled analysis have shown that HBOT was significantly effective in complete healing of diabetic foot ulcer (OR = 0.29; 95% CI 0.14–0.61; I^2^ = 62%) and reduction of major amputation (RR = 0.60; 95% CI 0.39–0.92; I^2^ = 24%). Although, it was not effective for minor amputations (RR = 0.82; 95% CI 0.34–1.97; I^2^ = 79%); however, less adverse events were reported in standard treatment group (RR = 1.68; 95% CI 1.07–2.65; I^2^ = 0%). Nevertheless, reduction in mean percentage of ulcer area and mortality rate did not differ in HBOT and control groups. This review provides an evidence that hyperbaric oxygen therapy is effective as an adjunct treatment measure for the diabetes foot ulcers. These findings could be generalized cautiously by considering methodological flaws within all studies.

## Introduction

Globally about 463 million people are living with diabetes, among them, three fourth (76.2%) are living in the middle, and a few (3.13%) are living in low-income countries. The International Diabetes Federation has anticipated the numbers of diabetes patients to raise to 700 million by 2045. Surprisingly, almost half of type-2 diabetes (DM2) adult patients are unaware that they are suffering from this disease. Moreover, 185.8 million undiagnosed diabetes are from middle-income countries^1^.

Most of the time DM2 remain completely asymptomatic for a long duration and by the time patient diagnoses they develop complications like neuropathy, retinopathy, metabolic disorders, diabetic foot ulcers (DFUs) which are later difficult to treat. Diabetic foot ulcer is defined as a presence of ulcer in the lower limb which is associated with neuropathy and/or peripheral arterial disease in a patient with diabetes^2^. Eventually, DFUs which are infected and multi-drug resistance (MDR), become non-healing and significantly contributes to amputations and mortality, specifically in the developing countries^3,4^.

Globally, every 30 s, a lower limb is lost, which is a major sequela of diabetes. The incidence rate of DFU in diabetes patient is 2%, (9.26 million) and this risk increases 17–60% with a previous history of DFU in next three years^5^; also, among them, half (4.63 million) of diabetes patients undergo lower limb amputation at some stage of their life^1^. Additionally, 28–51% DFU patients after the first amputation will have a higher probability of the second amputation in within five years^6^. Furthermore, a higher rate (28–77%) of mortality was reported between 90 days to 5 years of post-amputation in diabetic patients^7,8^, as well as reduction of survival rate at five-years were up to 55%, 34.4% after minor and major amputations respectively in diabetes patients with DFU^9^.

It has been evident that DFUs and subsequently amputations contribute to poor quality of life, the financial burden on the family & health care system, and increase the risk of early death^1,10^. Diabetic foot ulcer remains a big challenge for the patient as well as health care industry; this could be due to uneven health care facilities, lack of awareness, delayed referral, lack of specialised health care professionals^11^. In the line of standard treatment (ST) for DFUs, different modalities are available among them important are pressure relief, surgical debridement, antibiotics for the infection, and blood sugar control^12^.

Most of the time, foot ulcers in diabetes patients are infected with polymicrobial agents^13,14^, deprived body’s immune system^15^, and a high rate of antibiotics resistance are developed^4^, which results in non-healing ulcer. Importantly, chronic non-healing ulcers may not respond to the routine treatment and every patient with DFUs cannot be treated with surgical debridement that may necessitate alternative treatment modalities^16^. Hyperbaric oxygen therapy (HBOT) is one of the adjunct therapy^12^, which is used from decades to treat complex DFUs. In chronic wound, affected tissues become hypoxic, which hinders ulcer healing; hence oxygen plays a big role in chronic wound healing. In HBOT, the patient is kept in a chamber with 100% breathing oxygen and a higher atmospheric pressure greater than sea level (usually at 1.4 atmosphere absolute) for better clinical outcome^17,18^.

Many favourable physiological changes, such as augmented angiogenesis, improved collagen deposition, leukocyte activities, and decreased edema were reported among patients treated with HBOT^19^. HBOT helps in enhancing oxygen level in tissues to fasten the rate of ulcer healing process and further prevent amputations^20^. Despite these benefits with potential application for non-healing DFUs, HBOT remained under questionable therapy and kept as last option while treating DFUs.

**Figure 1:**
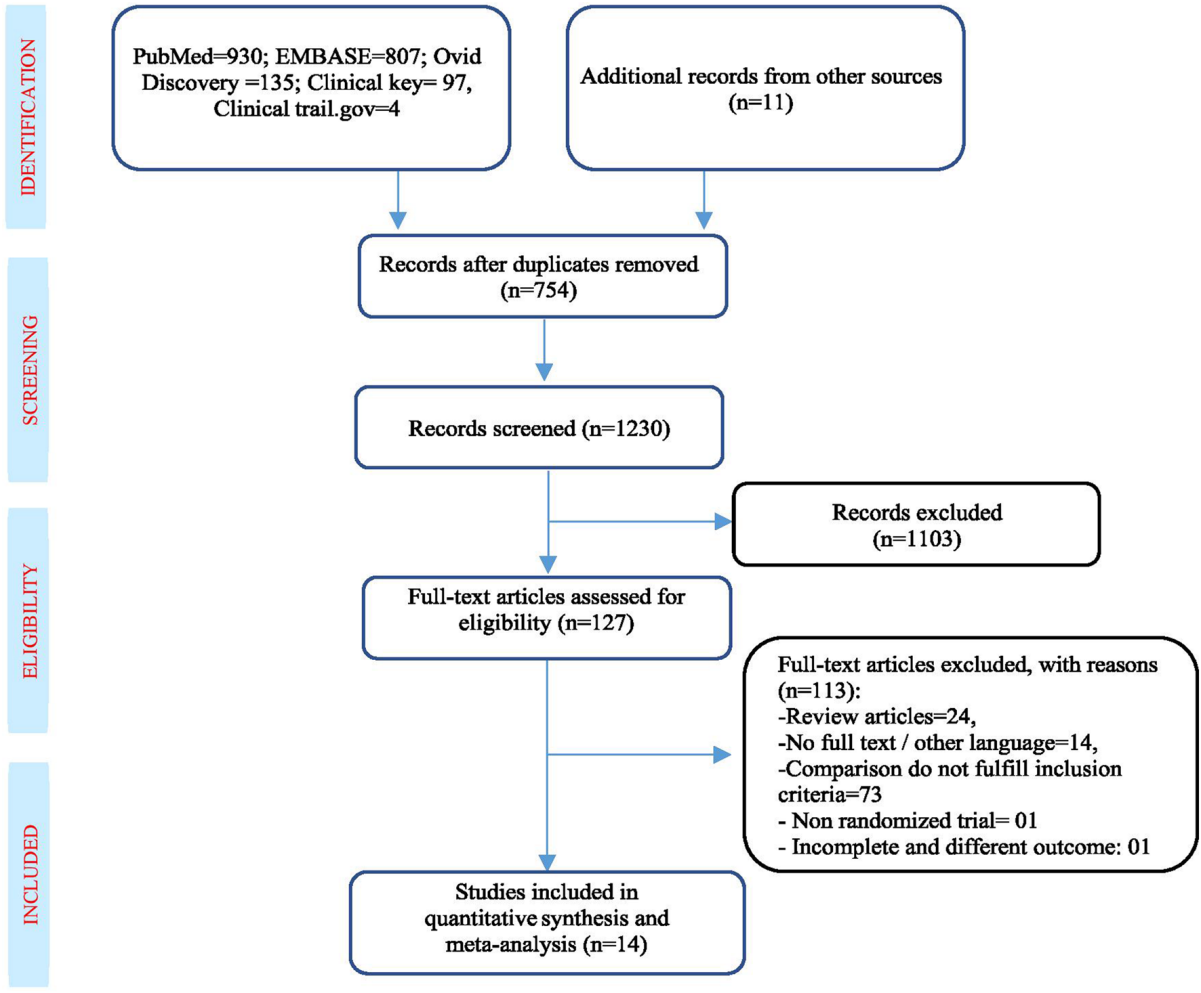
PRISMA flow diagram. From: Moher D, Liberati A, Tetzlaff J, Altman DG, The PRISMA Group (2009) Preferred Reporting Items for Systematic Reviews and Meta-Analyses: The PRISMA Statement. PLoS Med 6(7): e1000097. https://doi.org/10.1371/journal.pmed.1000097 (2009).

In 2015, Cochrane review by Kranke et al.^21^, concluded that a significant improvement in wound healing among patient treated by HBOT. Whereas, in a recent systematic review (SR) and meta-analysis (MA) by Brouwer et al.^22^, reported that HBOT reduces major amputation rate, but ineffective in wound healing; however, these researchers missed to include five RCTs, thus inferences drown in these reviews are not based on all the available evidences and may lack the strength. Moreover, these reviews inappropriately assessed quality of evidences and therefore inferences made in these reviews may be incomplete about the efficacy of HBOT in the management of non-healing DFUs. Therefore, keeping in mind methodological weaknesses of previous reviews, present methodologically sound SR&MA was aimed to provide most sound evidences about efficacy of the HBOT as an adjunctive therapy for the treatment of DFUs consisting all the new and old RCTs and CCTs (Fig. 1).

## Results

### Methodological quality of the studies

Studies were thoroughly reviewed and assessed for risk of bias by all the authors. All the domains of Cochrane risk assessment and details from methodology findings are given in Table 1. Random sequence generation was described in six studies^23–28^, whereas another seven trials^29–35^ were unclear about the process of random selection of study participants. One study^20^ mentioned in their methodology about randomization but not selected all the participant accordingly. Most of the trials had selection bias, five high biased^20,30,31,34,35^ and seven were unclear^24,26–29,32,33^.

**Table 1:** Characteristics of studies included in systematic review and meta-analysis.

Study, year, Design, and country	Number of Participants	Sample size determination	Mean Age ± SD (Years)	Gender Distribution M/F	Duration of DM (Years)	Hemoglobin A1c level	Wagner Grade									Interventional Protocol	Outcome Assessed
							HBOT					ST					
							I	II		III	IV	I	II	III	IV		
Abidia^29^, RCT, United Kingdom	Total = 18 HBOT = 9 ST = 9	Yes	HBOT = 72 ± 12.6	HBOT = 6/3	HBOT = 13 ± 9.9	–	0	8		–	–	1	7	–	–	90 min., 2.4 ATA, 5 days/week (30 sessions)	AR, RUS, CHU
			ST = 70 ± 6.6	ST = 3/6	ST = 10 ± 6.3	–											
Chaudhary^30^, RCT, India	Total = 40 HBOT = 20 ST = 20	No	HBOT = 45 ± 7.5	HBOT = 11/9	–	–	–	–		–	–	–	–	–	–	60 min., 2.4 ATA, 5 days/week (30 sessions)	HT, CHU, RUS
			ST = 43.8 ± 9.4	ST = 10/10	–	–											
Chen^23^, RCT, Taiwan	Total = 38 HBOT = 20 ST = 18	Yes	HBOT = 64.3 ± 13.0	HBOT = 10/10	HBOT = 13.7 ± 6.5	HBOT = 8.8 ± 2.0	–	–		–	–	–	–	–	–	120 min., 2.5 ATA, 5 days/week (20 sessions)	CUH, HRQoL
			ST = 60.6 ± 7.2	ST = 11/7	ST = 14.6 ± 6.6	ST = 8.3 ± 2.2											
Doctor^31^, RCT, India	Total = 30 HBOT = 15 ST = 15	No	HBOT = 56.2	HBOT = 12/3	HBOT = 9.8	–	–	–		–	–	–	–	–	–	45 min., 3 ATA, 2 days/week (04 sessions)	AR
			ST = 59.8	ST = 10/5	ST = 10.9	–											
Duzgun^24^, RCT, Turkey	Total = 100 HBOT = 50 ST = 50	Yes	HBOT = 58.1 ± 11.03	HBOT = 37/13	HBOT = 16.9 ± 8.24	HBOT = 8.7 ± 2.9	–	6		19	25	–	12	18	20	90 min., 2–3 ATA, 2 session/days followed by 1 session of following day (30–45 sessions)	AR, CHU
			ST = 63.3 ± 9.15	ST = 27/23	ST = 15.88 ± 5.56	ST = 8 ± 1.9											
Faglia^32^, RCT, Italy	Total = 68 HBOT = 35 ST = 33	Yes	HBOT = 61.7 ± 10.4	HBOT = 27/8	HBOT = 16 ± 10	HBOT = 9.3 ± 2.5	–	4		9	22	–	5	8	20	90 min., 2.2–2.4 ATA, 5 days/week	AR
			ST = 65.6 ± 9.1	ST = 21/12	ST = 19 ± 9	ST = 8.5 ± 2.3											
Fedorko^25^, RCT, Canada	Total = 103 HBOT = 49 ST = 54	Yes	HBOT = 61 ± 12	HBOT = 31/18	HBOT = 19.1 ± 11.5	HBOT = 8.27 ± 1.9	0	23		22	4	0	23	29	2	90 min., 244 kPa of pressure, 5 days/week (30 sessions)	AR, CHU, AE
			ST = 62 ± 12	ST = 38/16	ST = 12.4 ± 10	ST = 8.03 ± 2.05											
Kalani^20^, CCT, Sweden	Total = 38 HBOT = 17 ST = 21	No	HBOT = 54 ± 14	HBOT = 12/5	HBOT = 28 ± 12	HBOT = 7.1 ± 1.5	–	–		–	–	–	–	–	–	90 min., 2.5 ATA, 5 days/week (40–60 sessions)	AR, CHU, HT, M
			ST = 65 ± 11	ST = 18/3	ST = 26 ± 17	ST = 7.3 ± 1.4											
Kessler^26^, RCT, France	Total = 27 HBOT = 14 ST = 13	No	HBOT = 60.2 ± 9.7	HBOT = 10/4	HBOT = 18.2 ± 13.2	HBOT = 9.4 ± 2.4	–	–		–	–	–	–	–	–	90 min., 2.5 ATA, 5 days/week (20 sessions)	RUS, CHU
			ST = 67.6 ± 10.5	ST = 9/4	ST = 22.1 ± 13.1	ST = 8.1 ± 1.4											
Londahl^33^, RCT, Sweden	Total = 94 HBOT = 49 ST = 45	No	HBOT = 69	HBOT = 38/11	HBOT = 20 (1–63)	HBOT = 7.8	0	24		51	24	0	27	62	11	85 min., 2.5 ATA, 5 days/week (40 sessions)	AR, CHU, M
			ST = 68	ST = 38/7	ST = 23 (3–54)	ST = 8.1											
Ma^27^, RCT, China	Total = 36 HBOT = 18 ST = 18	No	HBOT = 59.8 ± 6.5	HBOT = 11/7	HBOT = 24.8 ± 16.9	–	4	4		10	–	5	6	7	–	90 min., 2.5 ATA, 5 days/week (20 sessions)	RUS
			ST = 60.4 ± 5.6	ST = 12/6	ST = 23.1 ± 16.6	–											
Perren^34^, CCT, Greece	Total = 26 HBOT = 13 ST = 13	No	–	HBOT = 10/3	HBOT ≤ 5 year = 6	–	2	2		9	–	2	2	9	–	120 min., ATA (unknown), 5 days/week (40 sessions)	RUS, UD
			–	ST = 10/3	> 5 years = 7 ST ≤ 5 year = 6 > 5 years = 7	–											
Salama^35^, RCT, Egypt	Total = 30 HBOT = 15 ST = 15	No	HBOT = 55.1 ± 7.5	HBOT = 12/3	HBOT = 20 ± 7.5	–	–	6		9	–	–	7	8	–	60 min., 2.5 ATA, 5 days/week (20–40 sessions)	CHU, RUH, AR
			ST = 57.7 ± 6.7	ST = 10/5	ST = 18 ± 8	–											
Santema^28^, RCT, Netherlands & Belgium	Total = 120 HBOT = 60 ST = 60	Yes	HBOT = 67.6 ± 10	HBOT = 51/9	HBOT = 16.6 ± 11.2	–	–	27		20	13	–	35	16	9	90 min., 2.4–2.5 ATA, 5 days/week (40 sessions)	AR, CHU, HT, M, QOL
			ST = 70.6 ± 11.2	ST = 46/14	ST = 18.8 ± 15.1	–											

*AE* adverse event, *AR* amputation rate, *CCT* controlled clinical trial, *CHU* complete healed ulcer, *DM*-diabetes mellitus, *HBOT* hyperbaric oxygen therapy, *QOL* quality of life, *HRQoL* Health Related Quality of Life, *HT* healing time, *M* mortality, *RCT* randomized controlled trial, *RUS* reduction in ulcer size, *SD* standard deviation, *ST* standard treatment, *UD* ulcer depth.

**Figure 2:**
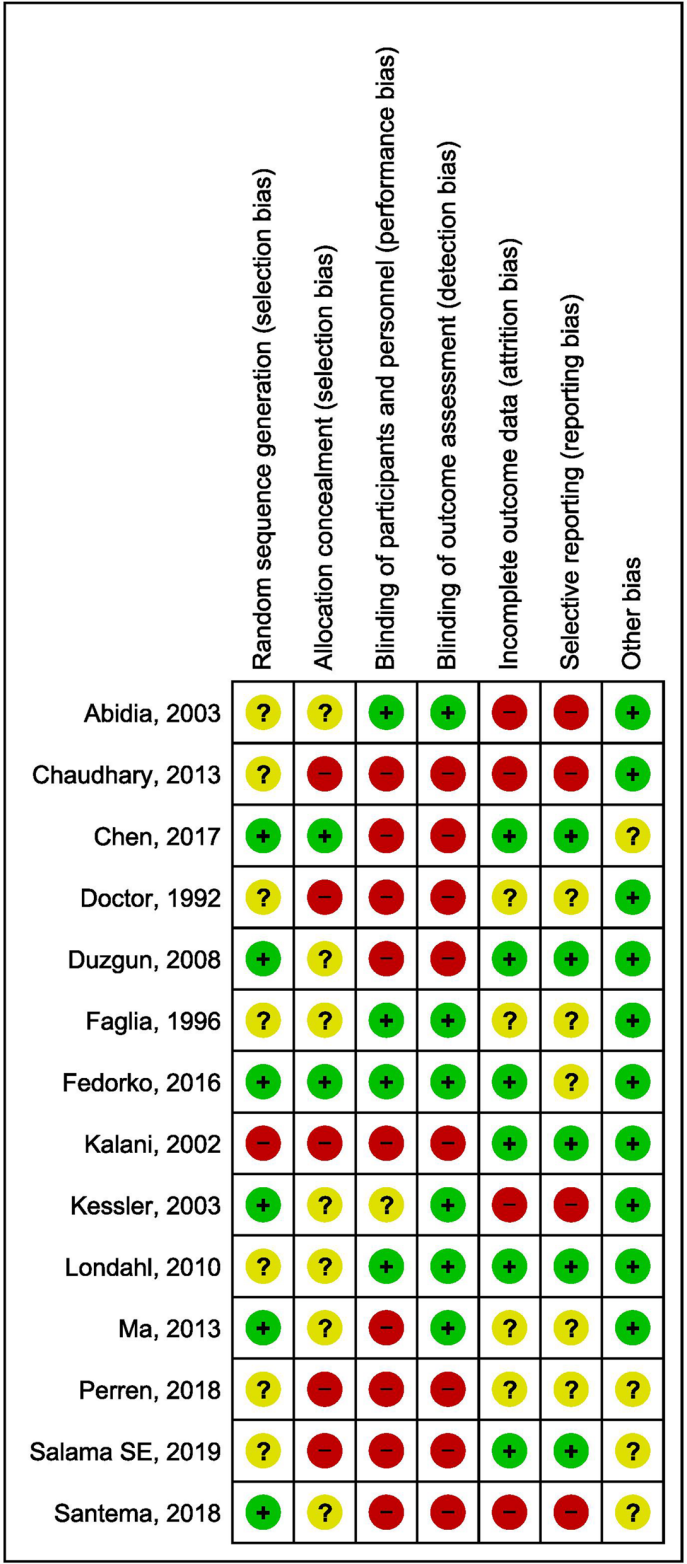
Risk of bias summary: review authors' judgements about each risk of bias item for each included study.

**Figure 3:**
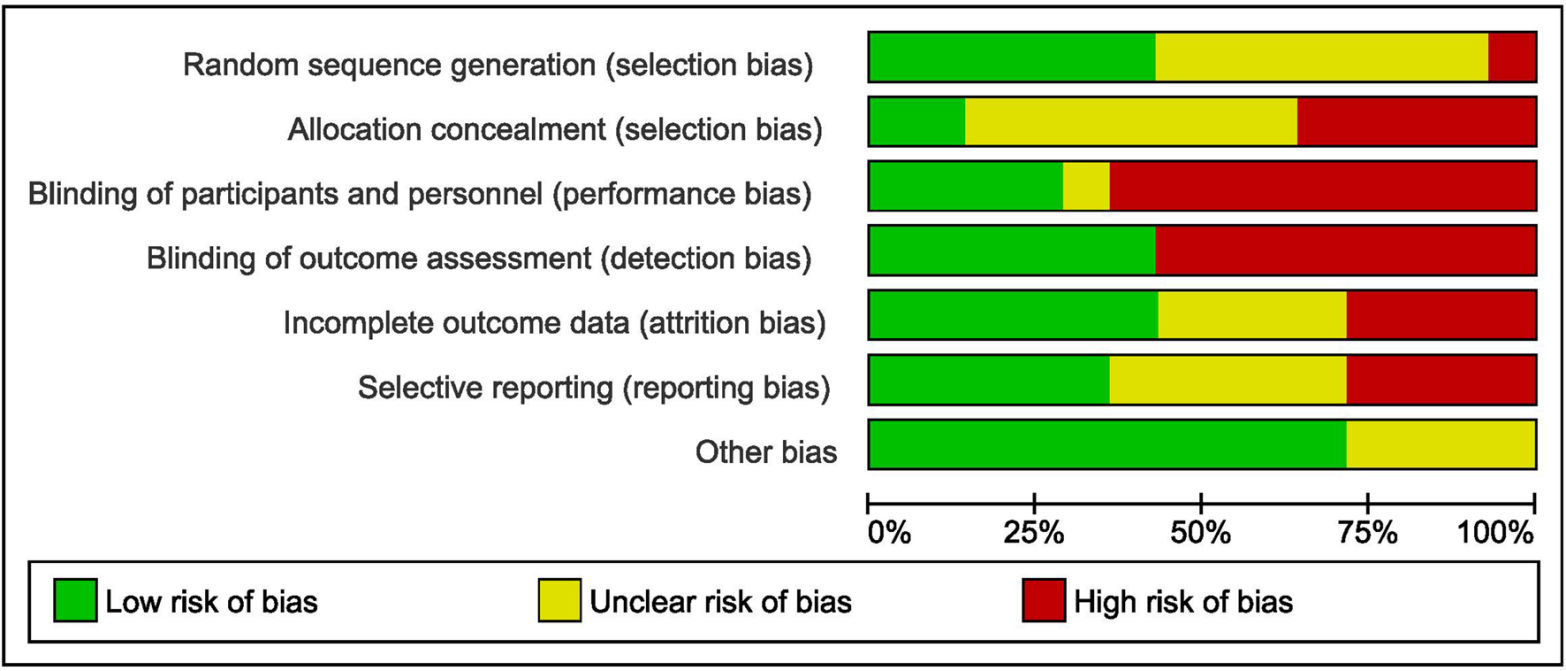
Risk of bias graph: review authors' judgements about each risk of bias item presented as percentages across all included studies.

Nine studies^20,23,24,27,28,30,31,34,35^ were at high risk for blinding of the participants while only one study^26^ was at unclear risk for the same. For outcome assessor blinding, it was reported that eight studies^20,23,24,28,30,31,34,35^ were at high risk and six studies^25–27,29,32,33^ were at low risk. Total four studies^26,28–30^ were at high risk for incomplete reporting or selective reporting bias and five^25,27,31,32,34^ were at unclear risk. In the other bias, only four studies^23,28,34,35^ were unclear, remaining all ten were in low risk category (Figs. 2, 3).

All the authors were taken in the loop, in case of any missing information from the study findings and after response has been received from corresponding authors of included studies, further decisions were made with the mutual consensus of all authors of this analysis.

### Characteristics of included studies

The characteristics of included studies are summarized in Table 1. There were total 14 studies which included 768 participants (384 in HBOT group and 384 in ST group). Out of 14 studies, there were twelve randomized controlled trials^23–33,35^, while two studies^20,34^ were controlled clinical trials. The haemoglobin A1c level was assessed in seven trails^20,23–26,32,33^. Eight RCTs^24,25,27–29,32,33,35^, and one CCT^34^ used the Wagner grading scale to grade the ulcers, a large number of patients were in grade-II and III. Majority of the studies^20,24–29,32^ had exposure of HBOT for 90 min, and in two studies^23,34^ the duration was 120 min, four trials^30,31,33,35^ used 45–85 min of time duration.

Eleven trails^20,23–30,33,35^ reported complete healed ulcer in their results. Most of the studies^20,23–25,27–29,31,32,35^ discussed amputation rate, whereas eight studies^24,25,28,29,31–33,35^ classified into minor and seven^25,28,29,31–33,35^ of them explained major amputations in their methodology. Only Duzgun et al.^24^ categorized amputation as distal and proximal to the metatarsophalangeal joint amputation. In the present study, meta-analysis for all group amputation rate was computed including the studies^23–25,28,29,31–33,35^ which had minor, major or undefined amputation as a study outcome. Adverse events were reported in seven studies^20,25,26,28,29,32,33^.

Only three studies^20,28,33^ discussed mortality during treatment. Reduction in mean percent ulcer size was reported by only three studies^25–27^, other studies^29,30,34,35^ which measured ulcer size but not appropriately reported in their results clearly; hence did not include in the meta-analysis.

Other outcomes of trails which are not included in the present study were: quality of life^34^, health-related quality of life^23^, healing time^20,30,34^, ulcer depth^34^, vascular intervention^28,33^, transcutaneous ulcer pressure^20,26–29,32,33^, and wound culture and sensitivity^20,23,27,30–32^.

### Complete ulcer healing

**Figure 4:**
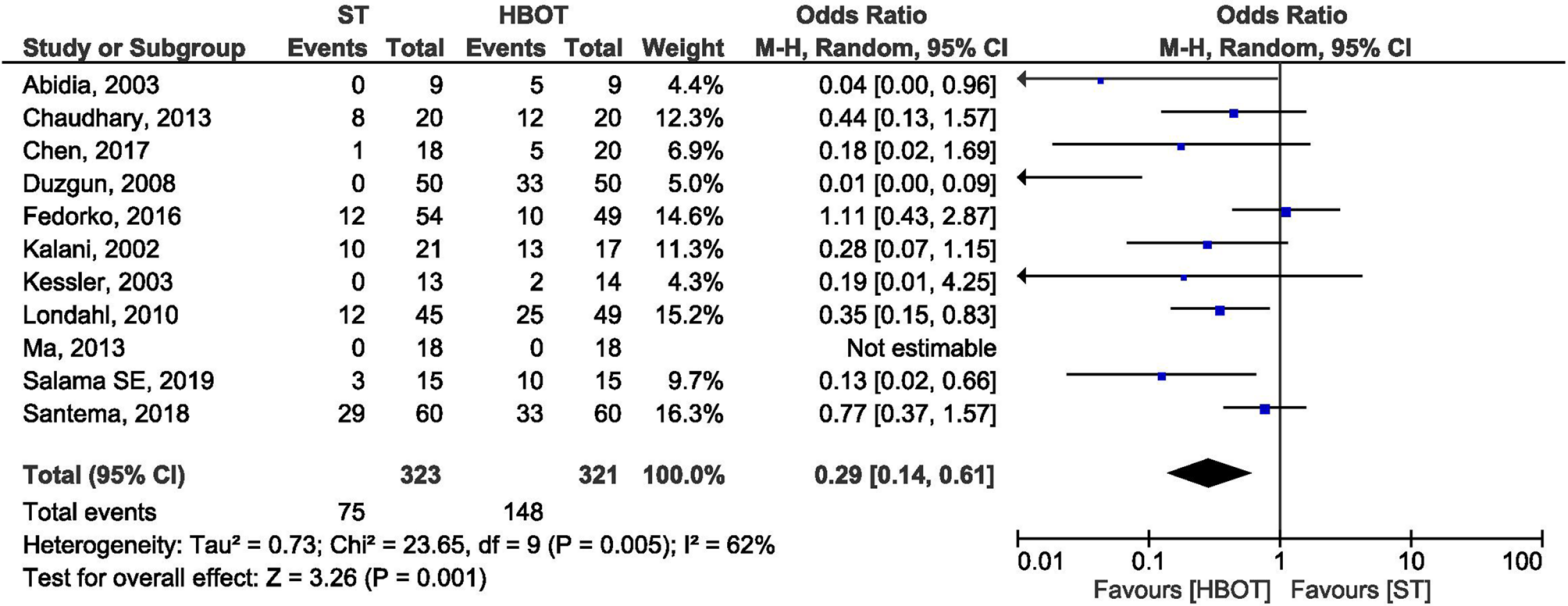
Forest plot of comparison: HBOT versus ST, Outcome: Complete Healed Ulcer.

Eleven studies were identified which reported complete ulcer healing with 644 patients randomized for HBOT and ST group (HBOT = 321; ST = 323). In the present meta-analysis, we included endpoint results which were different, from 14 days to 12 months to assess complete healed ulcer. Four trials^24,28,29,33^ reported complete ulcer healing at 12 months and seven below 12 months^20,23,25–27,30,35^. The number of complete healed ulcer after HBO therapy (148/321) were significantly higher compared to standard treatment (75/323) (OR = 0.29, 95% CI 0.14–0.61; I^2^ = 62%; *p* < 0.001; Fig. 4).

As the heterogeneity was high, we performed further analysis. Studies were classified into complete ulcer healing at 12 months and below 12 months. The findings of complete ulcer healing below 12 months (OR = 0.63; CI 0.39–1.02; I^2^ = 1%; *p* = 0.06) and, at 12 months (OR = 0.16; CI 0.03–0.82; I^2^ = 83%; *p* = 0.03).

### Major amputation

**Figure 5:**
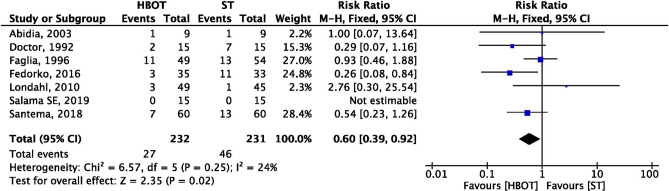
Forest plot of comparison: HBOT versus ST, Outcome: Major Amputation.

Major amputations rates were reported by seven trials comprising 232 patients in HBOT group versus 231 patients in ST group. Out of seven trials, only Abidia et al.^29^ did not define the part of the limb as major amputation but included in the outcomes. Four trials^28,31,33,35^ reported above ankle joint and two trials^25,32^ as above or below knee as major amputation. Pooled effect size from the fixed-effect model shows DFUs patients treated with HBOT had a significant lower major amputation rate as compared to ST and the difference was statistically significant as shown after analysis (HBOT 27/232 vs. ST 46/231; RR = 0.60; 95% CI 0.39–0.92; I^2^ = 24%; *p* = 0.02; Fig. 5).

### Minor amputations

**Figure 6:**
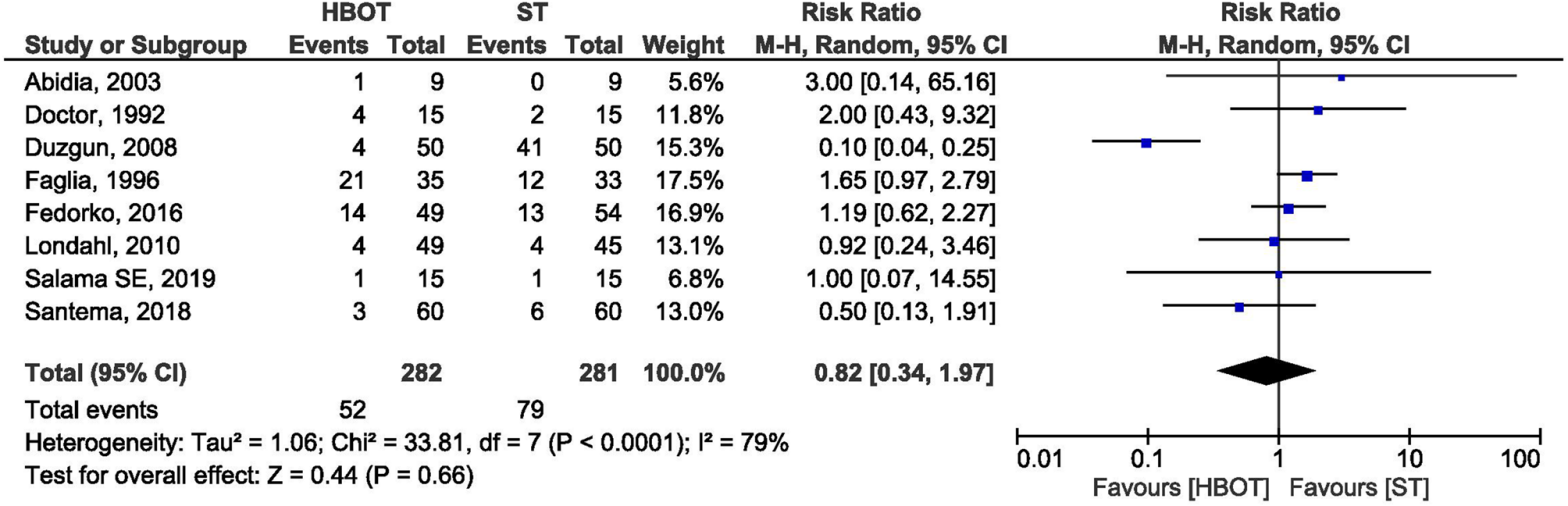
Forest plot of comparison: HBOT versus ST, Outcome: Minor Amputation.

Eight trails, including a total of 563 patients in HBOT (282) and ST group (281) reported minor amputation as a study outcome. Minor amputation was defined as below ankle or toe/forefoot by five trails^28,31–33,35^. While two trials^25,29^ did not define minor amputation. Whereas, Duzgun et al.^24^ classified as proximal and distal to metatarsophalangeal joint, which are included in the minor amputation category in present meta-analysis. Pooled result by a random effect model demonstrated that there was no significant difference in the numbers of minor amputations between HBOT and ST group. (RR = 0.82; 95% CI 0.34–1.97; I^2^ = 79%; *p* = 0.66; Fig. 6).

To explore the reason for the heterogeneity author excluded study performed by Duzgun et al.^24^ because of its extreme values in standard treatment group which was contradictory with other studies. Further analysis also indicates no significant different in the events of minor amputations between HBOT and ST group with 0% heterogeneity (RR = 1.32; CI 0.93–1.90; I^2^ = 0%; *p* = 0.12).

### All groups amputation

**Figure 7:**
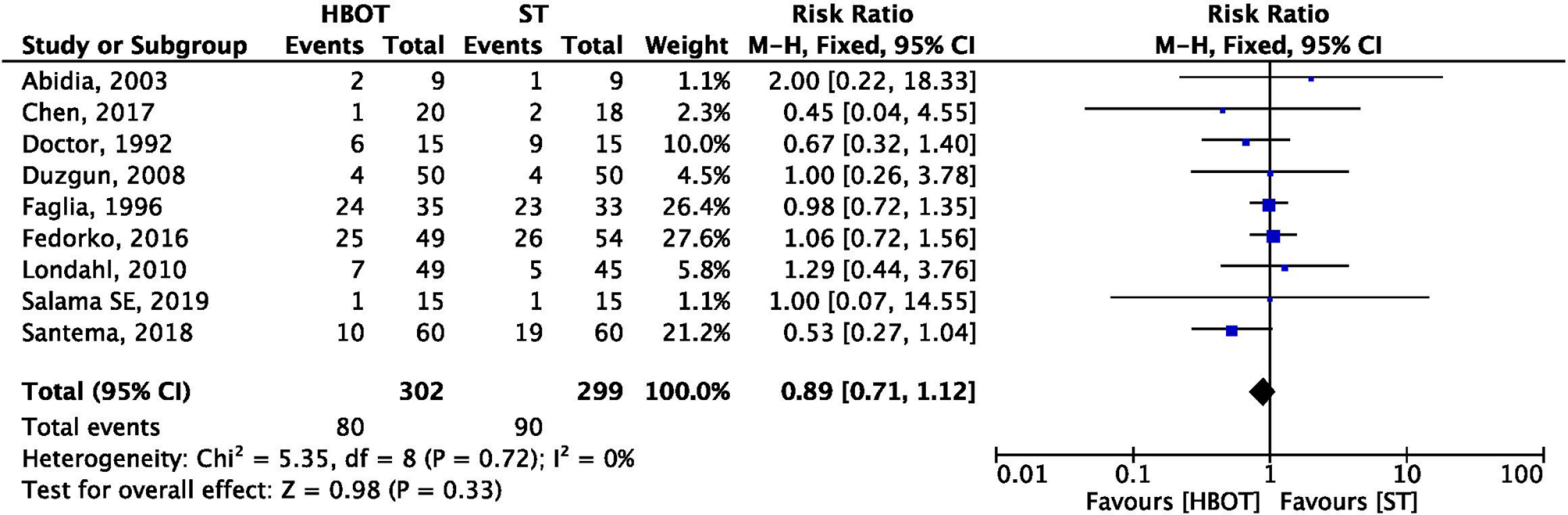
Forest plot of comparison: HBOT versus ST, Outcome: All groups amputation.

This variable was reported by nine trials, including 601 patients (HBOT-302; ST-299), reported that the rate of all groups amputation. We included nine trials which reported amputations, including minor and major amputations which were defined or undefined by the authors. Study by Chen et al.^23^ did not define but reported outcome as amputation, hence we have included that in all groups amputation rate. A forest plot in provided as Fig. 7 showing no statically differences between HBOT group and ST group from pooled results (RR = 0.89; 95% CI 0.71–1.12; I^2^ = 0%; *p* = 0.33).

### Adverse events

**Figure 8:**
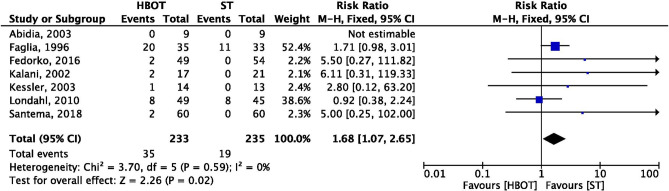
Forest plot of comparison: HBOT versus ST, Outcome: Adverse Event.

Forest plot (Fig. 8) shows the results of the meta-analysis of seven trials reported adverse events as one of the outcomes after interventions. The number of adverse events were high in the patients treated by hyperbaric oxygen therapy (35/233) than the standard treatment (19/235). After pooled proportion of result, it was reported that RR was 1.68 (95% CI: 1.07–2.65; I^2^ = 0%; *p* = 0.02) which was statistically significant.

### Mortality rate

**Figure 9:**
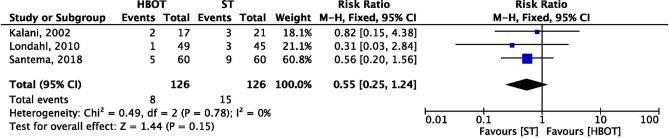
Forest plot of comparison: HBOT versus ST, Outcome: Mortality.

Three trails including a total of 252 patients (HBOT 8/126 and ST 15/126) reported mortality rate as an outcome measure. Maximum mortality was reported by Santema et al.^28^ 5/60 and 9/60 followed by HBO and standard therapy. Pooled data results by the fixed effect model showed that there was no significant difference (*p* = 0.15) in HBOT and ST group in mortality rate (RR = 0.55; 95% CI 0.25–1.24; I^2^ = 0%; Fig. 9).

### Reduction in ulcer area (%)

**Figure 10:**
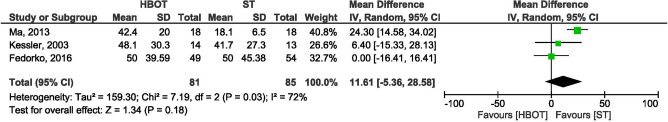
Forest plot of comparison: HBOT versus ST, Outcome: Mean ulcer area reduction (%).

Three trails^25–27^ included 166 patients (HBOT-81; ST-85) reported a reduction in mean percent of ulcer area as an outcome. Four trials^29,30,34,35^ reported reduction in the ulcer size, but results were incopmte, hence not included in meta-analysis. Forest plot shown in Fig. 10 reveals that pooled data from the completion of therapy found no significant differences (*p* = 0.18) in the percentage mean reduction ulcer size of patients in HBOT and ST group (Mean Difference (Md) = 11.61; 95% CI − 5.36 to 28.58; I^2^ = 72%).

### Publication bias

**Figure 11:**
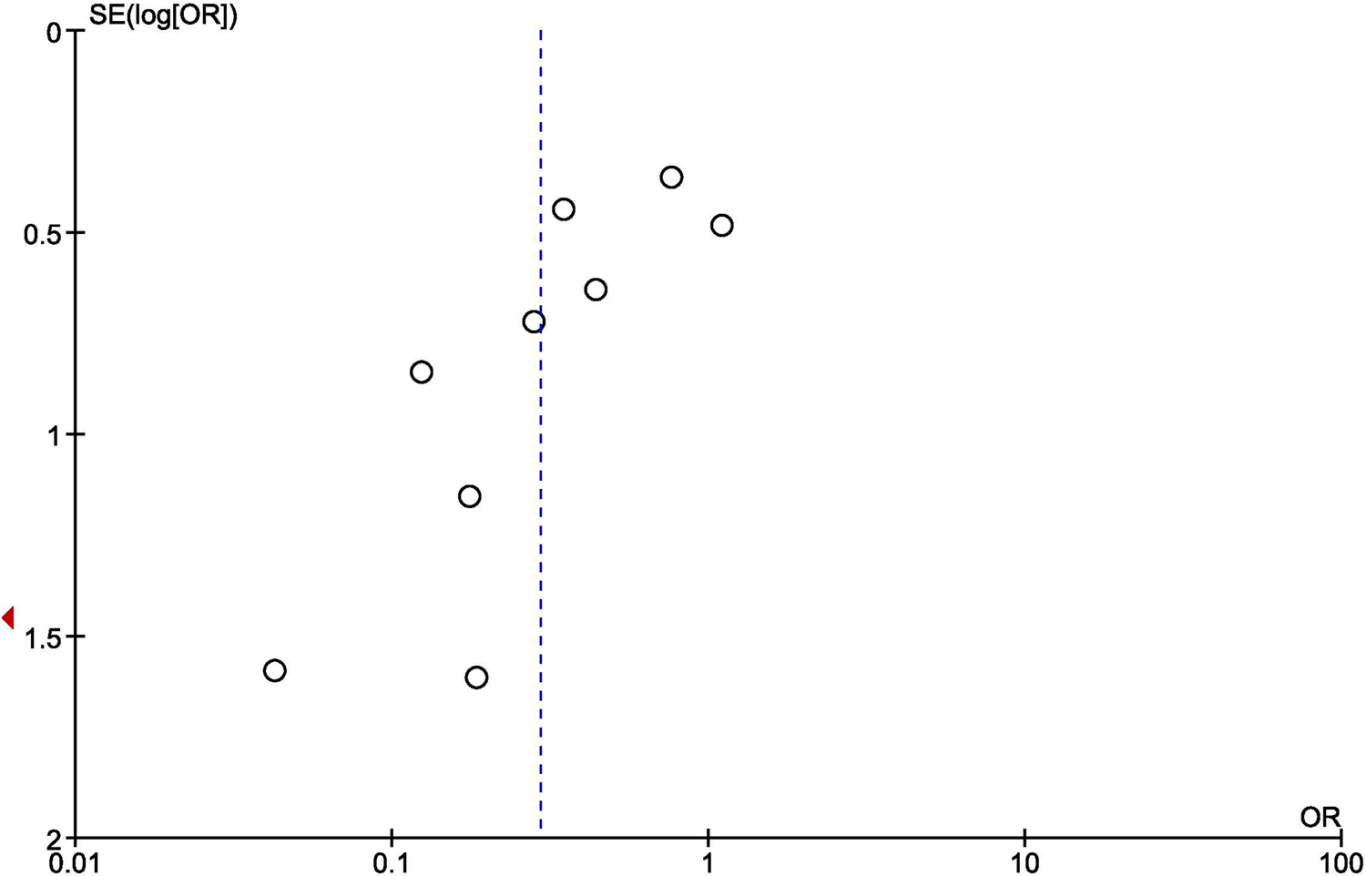
Funnel plot of comparison: Complete Healed Ulcer: HBOT versus ST.

A funnel plot was used to evaluate the publication bias for the selected outcomes of complete healed ulcer showed asymmetrical pattern, indicating a publication bias. (Fig. 11).

## Discussion

In this systematic review and meta-analysis, fourteen trials on efficacy of HBO therapy vs ST for the treatment of diabetic foot ulcers were included. This is the first SR and MA, which has included all the randomised control trials, and clinical control trials on efficacy of HBO therapy in the treatment of diabetic foot ulcers.

*Complete healed ulcer:* In our review, HBO therapy was found to be effective in the complete ulcer healing rate of DFUs among diabetic patients. Wound healing is a complex process in which oxygen plays an essential role. In chronic wounds, the level of oxygen declines and increasing oxygen level in wound tissues has shown better wound healing and less bacterial colonization^36^. In HBOT, human body receives 100% oxygen with higher atmospheric pressure than normal, which enhances the magnitude of oxygen in the human cell, and fasten wound healing process^20^. First randomised control trial^31^, and SR & MA^37^ also reported a higher rate of complete wound healing in patients with DFUs who received hyperbaric oxygen therapy than the standard treatment.

*Amputation rate:* The number of cases of minor, major and all group amputation were higher among standard group patients than HBOT group, but only in the major amputation rate there was a significance difference.

To find the potency of HBOT for minor, and all group amputation, our meta-analysis reported no significant between HBOT and ST. The cause of this discordant results is not readily ostensible, however, could be explicated by variability in the clinical profile of patients, comorbidities, differences in technique and duration of HBOT, O_2_ pressure and other possible confounding factors^22,25,38^.

Most of the trials^25,28,31,32^ in present MA had less number of major amputations in patients who underwent hyperbaric oxygen therapy then the standard treatment. Similarly in present MA for minor amputation, out of eight, five trials^24,28,29,31,35^ lower, one trail^33^ equal and two^25,32^ had high proportion of amputation in HBOT group. The heterogeneity was high (I^2^ = 79%) in the meta-analysis of minor amputation rate. Authors explored each trial and found that in a study Duzgun et al.^24^, number of surgical debridement cases were quiet high in ST group i.e. 24/50, while the number of patients underwent surgical debridement in HBOT group were only 4/50; these findings are although similar with other studies done by O'Reilly D et al.^39^, Rui L et al.^40^, and Kranke et al.^21^ in terms of positive outcomes that favours HBOT but still the variation in number of cases between both the groups were very much evident and that only contributed towards heterogeneity in the results.

Authors of this review would like to highlight one important point that it is not only HBOT alone but the total duration and number of sessions which could bring desired positive outcomes especially in reducing amputation rate among diabetic patients with DFUs. Three studies^28,33,35^ had reported less incidences of major as well as minor amputation and the only difference in the intervention which we could found was the number of HBOT sessions that was 40 in both studies. However, in all other trials there were 30 HBOT sessions were given to patients with DFUs and furthermore, higher incidences of minor and major amputation were reported in all these studies. This is the important observation made by authors and which could be considered while applying results of this evidence into practice or while conducting further RCTs on this area. The common associated factors that are well established for amputations among patients with non-healing DFU are: chronic arterial insufficiency^41^, neuroischemic foot^42^, poor glycaemic control^43^ and infection^44^. HBOT helps to accomplish physiological effects by declining ischemia at regional as well as local tissues. Therefore, HBOT helps to activate O_2_ dependent mechanism to enhance wound repair, production of stem cell in the bone marrow and improves host antimicrobial responses^20^.

*Adverse event*: Efficacy of any treatment also depends upon its adverse effects on the human being. In present analysis results from seven trials revealed that, the patient treated by HBOT experienced significantly higher rates of adverse events comparatively to those who were treated with standard treatment. In contrast to these results, a systematic review^40^ reported no difference in adverse events between the HBOT and ST group. Included studies had reported adverse events such as oxygen toxicity (O_2_ induced seizure)^33^; ocular effects, barotraumatic lesions, injury to ear^25,26,28,32,33^; hypoglycaemia^25,33^; and cataract^20,33^. There was a case of congestive heart failure reported by Fedorko^25^ followed by HBO therapy. The most common adverse effect associated with HBOT is barotrauma; it affects air-filled cavity in the human body (especially middle ear, lungs, and sinuses), which occurs due to compression. Mostly, barotrauma can be easily treated and recovered without therapeutic intervention. The major adverse effects are pulmonary barotrauma, injuries, or fire in the chamber; these are the rarest condition to happen^45^. These evidences are direction for future clinicians to establish safety protocol for patients while treating with hyperbaric oxygen therapy.

*Mortality*: The mortality rate was reported by three trials^20,28,33^ with higher rate in ST (15/126) than HBOT (8/126) group. In this meta-analysis, no significant difference was observed between HBOT and ST methods of treatment of patients with DFU. There were two cases of mortality, one due to multiorgan failure and another patient died due to progressive heart failure, and both the incidents did not occur during hyperbaric oxygen therapy^20^. The cause of deaths was, multiorgan failure^20,33^, progressive heart failure^20^, and gallbladder perforation followed by sepsis^28^ in HBOT group, but none of them were related to hyperbaric oxygen therapy. In consistent with the present study, a recent meta-analysis by Brouwer et al.^22^ evaluated the effect of HBOT on mortality reported similar results. They included three studies for meta-analysis, but one study by Abidia^29^ was not included for mortality as an outcome, furthermore Brouwer et al.^22^ in their SR & MA did not included a trail^33^ which was a latest publication on the topic.

*Mean ulcer area reduction*: In the diabetic wounds tissues, after HBO therapy a physiological initiate cellular and biochemical changes which support in wound healing^46,47^ which further results in raising in growth factors and fibronectin^48,49^ to fasten cellular proliferation, migration and formation of extracellular matrix molecules. In the present meta-analysis, there was no significant differences found in the reduction of mean percent of ulcer size in DFU patients treated with HBOT and standard treatment. Two trails^26,27^ included in meta-analysis evaluated ulcer size at baseline and two weeks of post-intervention of HBO therapy which had a higher reduction of mean percent of ulcer area than ST. Whereas, Fedorko et al.^25^ did not find significant differences between HBOT and ST groups at 12 weeks. In addition, a possible reason for non-significant results could be due to difference in the methods of measurement for ulcer size and, time duration of the assessment. However, other trails^29,30,34^, had shown a significant reduction of ulcer size among HBOT group at 4th, 6th, and 10th weeks of post-intervention. Present review is a current update on the topic of HBOT versus ST on which still many physicians have contradictory opinion and it was clearly evident that although we have evidences in hand but updated trials and review with high quality methodology is much needed to enhance better clinical practices. Further researches or strong RCTS with large sample size are required to employ the effectiveness of HBOT in reducing ulcer size in patients with DFUs.

## Limitations

In the present meta-analysis, there were some limitations. Out of 14 trials, only six studies^23–25,28,29,32^ performed sample size calculations, which could be an important weakness of trails. We observed the duration and techniques used in HBOT while treating patients were not uniform in most of the studies, which could have changed outcome. We tried to collect additional information which were not included in the publication, but none of the author responded to our quarries.

However, this meta-analysis is summarising the best available evidence for the specific group, i.e. patients with diabetic foot ulcers. Further, we strongly recommend large randomised control trials with rigour methodology to have further evidence to use of HBOT for the benefit of patients. Also, a team of clinical experts and researchers should plan a uniform and multicentric studies to explore the efficacy, safety and cost-utility of HBOT among patients could be of more use. Additionally, subgroup analysis will help for the scientific use of HBOT, so a large number of patients should be benefited from this therapy.

## Conclusion

This meta-analysis concludes that HBOT was associated with higher rates of complete healed DFUs and lower major amputation rates. however, it did not shown effect on reduction of minor amputation rate, all group amputation rate, mortality rate, and mean percent of ulcer size. While, adverse events were fewer in standard treatment group as compared HBOT. Hence, HBOT should be used with cautions for the treatment of DFUs and there is a great need of well-planned sufficiently larger size multicentric trials with a robust methodology to assess the efficacy and safety of HBOT as an adjuvant treatment for DFUs.

## Methods

We conducted this meta-analysis according to the Preferred Reporting Items for Systematic reviews and Meta-Analyses (PRISMA) statement and explained in the following section (Fig. 1).

### Systematic search

A systematic, in-depth search on randomised control trials (RCTs) and clinical control trials (CCTs) on effects of HBOT in patients with DFUs published in the PubMed, EMBASE, Clinical key, Ovid Discovery, ERMED, Clinical Trials.gov database and other sources was conducted. The search engine was filtered for time dated up to 15 September 2020; type of research were RCTs, and Controlled Clinical Trial on human. The main keywords used for search were "hyperbaric oxygenation therapy", "diabetic foot ulcer" and related MeSH terms. The search strategy details are given in Supplementary file-1. The reference list from selected studies was examined for additional trails and evidences that could have missed during primary searches.

### Study selection criteria

Studies were searched independently and screened potentially eligible studies by two investigators who read the title and abstract and related references, to select literature which requires further in a detailed examination. Whenever, there was any disagreement in the opinion of two investigators, then the third investigator was consulted to make a final decision for the study. Investigators also communicated the authors of the study, which required clarification. In the present study, the inclusion criteria were: (1) randomised controlled, and controlled clinical trial on humans, (2) patient with diabetic foot ulcers (DFUs) of any grade, (3) full-text articles in the English language, (4) studies which compare HBOT vs standard treatment (ST). Studies of reviews, editorials, meta-analyses, observational studies and studies which was unable to examine the results or without control group were excluded.

### Outcome measures

Two authors collected a predefined outcome from the studies, which includes, study characteristics, patients’ profile. The primary study outcomes: complete healed ulcer, major amputation rate, minor amputation rate, and rate of adverse events. In secondary outcomes: all group amputation rate, mortality rate, reduction in mean percent of ulcer area.

### Data extraction

After including studies in the review for meta-analysis, two independent primary reviewers extracted data in a data extraction form, and the third reviewer cross-checked all the data, which further enhances the authenticity of extracted data. The corresponding author was contacted in case of any queries related to study findings.

### Quality assessment

Four reviewers reviewed all included studies, and they used the Cochrane Collaboration approach for the assessment of risk bias. Two primary reviewers assessed for randomization bias, allocation concealment, blinding of participants and assessor, incomplete outcome data and other bias (Fig. 2). All studies were reported as low risk, high risk and unclear risk for its biasness towards each component. In case a study reported low risk for all domains of risk of biasness, it was considered to be of good quality and vice-versa. In case of any contrary opinion between primary reviewers for risk bias, third and fourth reviewers did a thorough assessment of the study and conclusions were made with mutual consensus. A subjective report on the Risk of Bias is given in Supplementary file-2.

### Data analysis

We used Cochrane RevMan software (version 5.3)^50^ for statistical analysis of the pooled data. In all the analysis, a heterogeneity test was computed using the I^2^ statistics, which quantify the level of inconsistency in the results. A fixed effect model was considered to compare between the groups with heterogeneity (I^2^) below 50%, and if value > 50% a random effect model was used. Also, we examined the meta-analysis by removing one study at a time to check if any individual study affecting the heterogeneity. In the results, risk ratio (RR) was used for the assessment of negative outcomes like major amputation, minor amputation, all group amputation, adverse event and mortality; odds ratio was preferred in evaluation of complete ulcer healing which is a positive outcome. Further, mean difference (MD) for continuous variable were used with confidence interval (CI) at 95%. In order to identify the potential threat of publication bias, a funnel plot was drawn, which indicates the risk of publication bias (Fig. 11).

## Supplementary Information

Supplementary Information 1.

Supplementary Information 2.
