# Supplementary material for: Efficacy of hyperbaric oxygen therapy for diabetic foot ulcer, a systematic review and meta-analysis of controlled clinical trials
Source: Sci Rep. 2021 Jan 26;11:2189. doi: 10.1038/s41598-021-81886-1 (PMC7838311; doi:10.1038/s41598-021-81886-1)
Supplement: Supplementary file 1 — Supplementary Information 1. [file 41598_2021_81886_MOESM1_ESM.docx]

**Search Strategy Details**

| **Pubmed Central Search Strategy**  ("Diabetic Foot"[Mesh]) AND "Foot Ulcer"[Mesh]  ("Diabetic Foot"[Mesh]) AND "Hyperbaric Oxygenation"[Mesh]  (((("Foot Ulcer"[Mesh])) OR "Diabetic Foot"[Mesh]) AND "Hyperbaric Oxygenation"[Mesh]) OR "Placebos"[Mesh]  ((("Diabetic Foot"[Mesh]) OR "Foot Ulcer"[Mesh]) AND "Atmosphere Exposure Chambers"[Mesh]) OR "Atmospheric Pressure"[Mesh]  ((("Diabetic Foot"[Mesh]) AND "Atmospheric Pressure"[Mesh]) OR "Atmosphere Exposure Chambers"[Mesh]) OR "Hyperbaric Oxygenation"[Mesh]  ((((("Diabetic Foot"[Mesh]) OR "Foot Ulcer"[Mesh]) OR "Wound Healing"[Mesh]) AND "Hyperbaric Oxygenation"[Mesh]) OR "Standard of Care"[Mesh]) OR "Reference Standards"[Mesh]  (("Diabetic Foot"[Mesh]) AND "Aftercare"[Mesh]) AND "Hyperbaric Oxygenation"[Mesh]  (((("Diabetic Foot"[Mesh]) OR "Foot Ulcer"[Mesh]) AND "Wound Healing"[Mesh]) AND "Hyperbaric Oxygenation"[Mesh]) AND "Aftercare"[Mesh] |
| --- |
| **Ovid Embase Search Strategy**  'diabetic foot infection'/exp  'hyperbaric oxygen therapy'/exp  'hyperbaric chamber'/exp  'diabetic foot infection'/exp AND 'hyperbaric oxygen therapy'/exp  ‘conservative treatment’/exp OR 'hyperbaric oxygen therapy'/exp  'foot ulcer'/exp OR ‘placebo effect’/exp OR ‘hyperbaric chamber’/exp  'conservative treatment'/exp AND 'high pressure processing'/exp AND 'chronic wound'/exp  'diabetic foot infection'/exp AND 'hyperbaric oxygen therapy' AND 'conservative treatment'/exp  'foot ulcer'/exp OR 'chronic wound'/exp OR 'diabetic foot infection'/exp OR 'hyperbaric oxygen therapy'/exp  'conservative treatment'/exp OR 'placebo effect'/exp AND 'hyperbaric oxygen therapy'/exp AND 'foot ulcer'/exp OR |
| **Scopus**  high AND pressure AND oxygen AND therapy AND versus AND standard AND treatment  hyperbaric AND oxygen AND therapy AND in AND treatment AND of AND diabetic AND foot  hyperbaric AND oxygen AND therapy AND versus AND standard AND treatment AND for AND diabetic AND foot  AND ulcer AND treatment  oxygen AND therapy OR standard AND treatment AND diabetic AND foot OR foot AND ulcer OR chronic AND  wound  diabetic AND foot OR chronic AND wound AND hyperbaric AND oxygen AND therapy AND versus AND standard  AND routine AND treatment |

**PICO Framework**

| **Population** | Adult diabetes patients with non-healing lower limb ulcer or diabetic foot ulcers (DFUs) of any grade |
| --- | --- |
| **Intervention** | Hyperbaric oxygen therapy (any duration and number of sessions) for treatment or re-epithelialization of diabetic foot ulcer. |
| **Comparison** | Standard treatment with wound care, infection control, debridement, prescription for off-loading and advanced wound care. |
| **Outcome** | **Primary Outcome:** Complete healed ulcer, major amputation rate, minor amputation rate, and rate of adverse events.  **Secondary Outcome:** All group amputation rate, mortality rate, reduction in mean percent of wound area. |
