# Supplementary material for: Efficacy of hyperbaric oxygen therapy for diabetic foot ulcer, a systematic review and meta-analysis of controlled clinical trials
Source: Sci Rep. 2021 Jan 26;11:2189. doi: 10.1038/s41598-021-81886-1 (PMC7838311; doi:10.1038/s41598-021-81886-1)
Supplement: Supplementary file 2 — Supplementary Information 2. [file 41598_2021_81886_MOESM2_ESM.docx]

**Summary of Risk of Bias with rational**

| **Study** | **Randomization** | **Allocation** | **Participant & Personnel Blinding** | **Outcome Blinding** | **Incomplete Outcome Data** | **Incomplete Outcome Data addressed** | **Selective Reporting** |
| --- | --- | --- | --- | --- | --- | --- | --- |
| **Abidia, 2003** | **Unclear**  Randomized to group but incomplete explanation about standardized method of randomization | **Unclear**  Opaque sealed and sequentially numbered envelops were not used | **Low**  All patients and their relatives were blinded to treatment | **Low**  Medical assessors were blinded to treatment | **High**  02 patients withdrew from the study | **High**  02 patients (1 from experimental and 1 from control withdrew during the course of study and final analysis did not include their details) | **Unclear**  Almost all the outcomes are reported but HADS for depression and SF-36 for general health , vitality domain are only reported in terms of p-value and other details are not mentioned |
| **Chaudhary, 2013** | **Unclear**  Randomized but process or method was not explained | **High**  Allocation concealment was not performed | **High**  Participants were not blinded | **High**  No mention about blinding of outcome assessors | **High**  15 patients withdrew from the study | **High**  No inclusion of 15 patients in final analysis or results | **Low**  All the study outcomes are reported |
| **Chen, 2017** | **Low**  Randomized with standardized method | **Low**  Allocation done in opaque sealed sequentially numbered envelop | **High**  Participants were aware of their treatment as they discussed treatment with physician or research team | **High**  Outcome assessors were not blinded in this research trial | **Low**  Complete outcome data reporting | **Low**  Complete outcome data reporting | **Unclear**  Almost all the outcomes are reported but HRQOL and SF-36 are only reported in terms of p-value and other details of total score are not reported |
| **Doctor, 1992** | **Unclear**  Process of randomization not explained | **High**  Not performed as it was not mentioned anywhere | **High**  Participants were not blinded as not statement was given | **High**  No mention about who all were involved in outcome assessment and it seems to be not blinded | **Unclear**  It was not mentioned that how many withdrew during study | **Unclear**  No clear documentation of whether results included who withdrew from the study or not | **Low**  All the study outcomes were clearly reported |
| **Duzgun, 2008** | **Low**  Process of randomization followed properly | **Unclear**  Not mentioned about opaque sealed envelope was not used | **High**  Participants blinding were not mentioned | **High**  Not mentioned about outcome assessor blinding | **Low**  No participants withdrew from study as analysis | **Low**  Complete outcome data reporting | **Low**  All the study outcomes were clearly reported |
| **Fedorko, 2016** | **Low** | **Low** | **Low** | **Low** | **Low** | **Unclear**  Although it was mentioned that missing data was carried out with last observation but it was not clear that it was same for participants who withdrew from study | **Low** |
| **Fagilia, 1996** | **Unclear**  Randomization done on hospital identified data and process not explained | **Unclear**  Allocation was mentioned but there was no mention that how it was done | **Low** | **Low** | **Unclear**  Initially there were 34 participants in each group but later 35 in HBOT and 33 in non-HBOT and no explanation for that | **Unclear**  No clear justification for analysis of data with 35 in HBOT and 33 in non-HBOT | **Low** |
| **Kalani, 2001** | **High**  Randomization not done for all participants | **High**  Not mentioned anywhere | **High**  Participants’ blinding were not done | **High**  There was no mention of outcome assessor blinding | **Low**  Complete reporting of those who died | **Low**  Analysis include information of all participants who withdrew | **Low** |
| **Kessler, 2003** | **Low** | **Unclear**  No clear mention of allocation concealment | **Unclear**  It was mentioned that conventional additional treatment was given to both the group which is indicative of blinding but not clear | **Low**  Physician were blinded to the type of treatment | **High**  01 patient withdrew from the study | **High**  Final analysis did not include patients who withdrew from the study | **Low** |
| **Londahl, 2010** | **Unclear**  Although done in a block of 10 but did not explain the process | **Unclear**  Not mentioned about whether envelops were opaque or sequentially numbered | **Low** | **Low** | **Low** | **Low** | **Low** |
| **Ma, 2013** | **Low** | **Unclear**  Not clearly mentioned | **High**  Blinding of participants were not performed | **Low**  Physicians were blinded to the study | **Unclear**  Participants withdrew from the study | **Unclear**  No inclusion of all patients in final analysis | **Low** |
| **Perren, 2018** | **Unclear**  Randomization process was not explained | **High**  No mention of allocation concealment | **High**  Blinding of participants not mentioned | **High**  Blinding of outcome assessors not mentioned | **Unclear**  Not clearly mentioned about participants who withdrew from study | **Unclear**  Analysis did not include complete details of participants who have withdrawn | **Unclear**  Incomplete data reporting |
| **Santema, 2018** | **Low** | **Unclear**  Not clearly mentioned | **High**  Blinding of participants not mentioned | **High**  Blinding of outcome assessors not mentioned | **High**  11 who withdrew from intervention | **High**  Final analysis did not include information of those who withdrew from the study | **Unclear**  Incomplete data reporting |
